# Pregabalin in fibromyalgia - responder analysis from individual patient data

**DOI:** 10.1186/1471-2474-11-150

**Published:** 2010-07-05

**Authors:** Sebastian Straube, Sheena Derry, R Andrew Moore, Jocelyn Paine, Henry J McQuay

**Affiliations:** 1 Department of Occupational and Social Medicine, University of Göttingen, Waldweg 37 B, D-37073 Göttingen, Germany; 2 Pain Research and Nuffield Department of Anaesthetics, University of Oxford, John Radcliffe Hospital, Level 6 West Wing, Oxford, OX3 9DU, UK; 3 Spreadsheet Factory, 23 Stratfield Road, Oxford OX2 7BG, UK

## Abstract

**Background:**

Population mean changes are difficult to use in clinical practice. Responder analysis may be better, but needs validating for level of response and treatment duration. A consensus group has defined what constitutes minimal, moderate, and substantial benefit based on pain intensity and Patient Global Impression of Change scores.

**Methods:**

We obtained individual patient data from four randomised double blind trials of pregabalin in fibromyalgia lasting eight to 14 weeks. We calculated response for all efficacy outcomes using any improvement (≥ 0%), minimal improvement (≥ 15%), moderate improvement (≥ 30%), substantial improvement (≥ 50%), and extensive improvement (≥ 70%), with numbers needed to treat (NNT) for pregabalin 300 mg, 450 mg, and 600 mg daily compared with placebo.

**Results:**

Information from 2,757 patients was available. Pain intensity and sleep interference showed reductions with increasing level of response, a significant difference between pregabalin and placebo, and a trend towards lower (better) NNTs at higher doses. Maximum response rates occurred at 4-6 weeks for higher levels of response, and were constant thereafter. NNTs (with 95% confidence intervals) for ≥ 50% improvement in pain intensity compared with placebo after 12 weeks were 22 (11 to 870) for pregabalin 300 mg, 16 (9.3 to 59) for pregabalin 450 mg, and 13 (8.1 to 31) for pregabalin 600 mg daily. NNTs for ≥ 50% improvement in sleep interference compared with placebo after 12 weeks were 13 (8.2 to 30) for pregabalin 300 mg, 8.4 (6.0 to 14) for pregabalin 450 mg, and 8.4 (6.1 to 14) for pregabalin 600 mg. Other outcomes had fewer respondents at higher response levels, but generally did not discriminate between pregabalin and placebo, or show any dose response. Shorter duration and use of 'any improvement' over-estimated treatment effect compared with longer duration and higher levels of response.

**Conclusions:**

Responder analysis is useful in fibromyalgia, particularly for pain and sleep outcomes. Some fibromyalgia patients treated with pregabalin experience a moderate or substantial pain response that is consistent over time. Short trials using 'any improvement' as an outcome overestimate treatment effects.

## Background

Fibromyalgia is surrounded by controversy regarding its aetiology and its status as a valid disease entity. Genetic and neurobiological evidence now exists to support differences between fibromyalgia patients and controls [1]. Candidate biomarkers identifying susceptible individuals or indicating disease activity are emerging, [2] along with a better understanding of outcomes in clinical trials [3].

Fibromyalgia is characterised by widespread pain for longer than three months with pain on palpation at 11 or more of 18 specified tender points [4]. Sleep disturbance, depression, and fatigue often complicate the clinical picture [5]. Fibromyalgia is common, occurring in 1-2% of the population, more often in women than men, [6-8] and often with profound impact on activities of daily living and productivity [9,10].

It is increasingly recognised that medicines typically provide a good response in half or fewer of patients treated [11,12]. This is true in acute pain, [13] neuropathic pain, [14-16] migraine, [17] and osteoarthritis [18,19].

Here we present an analysis of the efficacy of pregabalin in fibromyalgia using individual patient data from four randomised, double blind, placebo controlled trials (RCTs). With this analysis we aimed to identify which outcomes were appropriate for a responder analysis based on the Initiative on Methods, Measurement, and Pain Assessment in Clinical Trials (IMMPACT) consensus statement on interpreting changes in chronic pain clinical trial outcomes [20]. This suggested that for pain, a minimally important improvement was a 10-20% decrease in pain intensity, a moderately important improvement a decrease of 30% or more, and a substantial improvement a decrease of 50% or more. It also suggested that responses in Patient Global Impression of Change of minimally improved, much improved, and very much improved would also constitute minimally important, moderately important, and substantial improvements.

IMMPACT defined response in dimensions other than pain, including physical and emotional functioning, as well as global rating of improvements. In theory, any measurement on any scale could be used for a responder analysis, with a wide range of possibilities of what constitutes a responder. The use of change from baseline, with several different levels of response, should allow an assessment of the utility of both the scale, and the level of response. Utility can be assessed by the occurrence of statistically or clinically significant differences between active therapy and placebo for a particular scale, especially if there appears to be a dose response. The absence of a significant difference between an effective therapy and placebo at all levels of response would be an indication that that particular scale lacks utility for measuring response in a particular circumstance.

The particular circumstance of fibromyalgia is interesting because many different measurements are made using different scales, allowing different scales and levels of response to be examined.

## Methods

Pfizer Inc provided Excel files containing individual patient data from four multi-centre clinical phase 2/3 or phase 3 RCTs of pregabalin (Lyrica) in the treatment of fibromyalgia that were conducted in the USA and other countries and were completed by July 2008 (trials 105, [21] 1056, [22] 1077, [23] 1100 [24]). Pfizer Inc also provided PDF files of the corresponding company clinical trial reports. A trial of enriched enrolment randomised withdrawal design ("FREEDOM trial", 1059 [25]) was not included in our analysis because it was fundamentally different [26].

Trial patients were at least 18 years old. Women were not pregnant or lactating, and either postmenopausal, surgically sterilised, or using contraception. Important exclusion criteria were: severe pain due to other conditions, rheumatic diseases other than fibromyalgia, active infections, untreated endocrine disorders, severe depression, active malignancy, being immunocompromised, other severe acute or chronic medical or psychiatric conditions, or laboratory abnormalities. Trial patients had to fulfil ACR criteria for fibromyalgia and have pain scores of ≥ 40 mm on the 100 mm visual analogue scale (VAS) after stopping any relevant pain or sleep medication. Patients were randomised to receive pregabalin (150 mg, 300 mg, 450 mg, or 600 mg per day), or placebo, predominantly with a 2-week dose escalation phase followed by fixed dosing for up to 14 weeks of total trial duration.

We calculated the proportion of patients achieving reductions in pain scores of any improvement (≥ 0%), ≥ 15%, ≥ 30%, ≥ 50%, and ≥ 70% compared to baseline pain scores between weeks 1-12. Sleep improvement was calculated in an analogous manner from weekly averages of sleep quality scores. Improvements in end of trial outcomes (Hospital Anxiety and Depression Scale [HADS], Fibromyalgia Impact Questionnaire [FIQ], Short Form 36 [SF-36] domains, Multidimensional Assessment of Fatigue [MAF] global index, Patient Global Impression of Change [PGIC], Medical Outcomes Study [MOS] Sleep Disturbance, and MOS 9-item Sleep Problem Index), were calculated by comparing data at the trial endpoints with baseline data and calculating the percentage improvement with the individual baseline score set as 100%. We chose levels of improvement for non-pain outcomes also at the above-mentioned cut-points in order to allow ready comparison with pain as an outcome, although it has to be kept in mind that those cut-points do not necessarily have the same clinical relevance for non-pain outcomes as they do for pain (where they have been validated).

The following two rules were applied to the data set to handle missing data.

• For patients who did not drop out, only actual measured values were used for calculations. Last observation carried forward was not used except where no other data were available (for end of trial outcomes in trial 105 and for HADS outcomes from all trials).

• From discontinuation day forward patients were assigned 0% improvement.

A responder is then defined as any patient who achieves at least the predefined level of change specified or greater. For example, a patient with exactly 50% pain relief and a patient with 57% pain relief would both be counted as responders at the 50% level.

Trial quality was assessed using the Oxford Quality Scale [27]. Validity was scored using the Oxford Pain Validity Scale [28]. The minimum requirement for inclusion in this responder analysis was that trials had to be both randomised and double blind.

Calculations of responder rates and numbers needed to treat (NNT) were performed independently of Pfizer using a spreadsheet consultancy (Spreadsheet Factory -- http://www.spreadsheet-factory.com) run by one of the authors (Jocelyn Paine). Response data were pooled and used in an intention-to-treat analysis including all randomised patients who received at least one dose of trial drug. We calculated the number and percentage of responders for each level of response (≥ 0%, ≥ 15%, ≥ 30%, ≥ 50%, and ≥ 70% improvement compared to baseline pain scores), pregabalin dose (300 mg, 450 mg, or 600 mg per day), and time point (per week of trial or at end of trial, as detailed in the figures and tables). NNTs were calculated with 95% confidence intervals by the method of Cook and Sackett, [29] using the pooled number of observations. NNTs were not calculated when statistical significance was not achieved; in this circumstance NNTs can approach infinity (100/absolute risk difference), with one of the confidence limits being negative. Only data from trials that included a particular pregabalin dose were used for calculations for that dose; only the placebo data from the specific trials which included that specific dose were used in each dosing comparison. The intention was to analyse data only where there were at least 200 patients in at least two trials [30].

For responder analysis to be useful we hypothesised that its should produce stepped reductions in the percentage of patients responding with increasing level of response, a significant difference between pregabalin and placebo in the number of responders at a particular level, and a trend towards lower (better) NNTs at higher doses of pregabalin, given that pregabalin has been shown in randomised trials and meta-analysis to be effective in fibromyalgia, with higher doses being more effective and with more adverse events [31]. Any scale without these features would be unlikely to have any utility for a responder analysis in fibromyalgia.

## Results

### Patient and trial characteristics

In the four trials 2757 patients aged between 18 and 82 years were treated with pregabalin or placebo. More than 90% were women. One trial lasted 8 weeks (trial 105); the others lasted 13 or 14 weeks. All trials were of high quality and validity, scoring 5/5 on the Oxford Quality Scale and 16/16 on the Oxford Pain Validity Scale. Pregabalin doses of 300 mg (685 patients) and 450 mg (687 patients) were used in all four trials, 600 mg (564 patients) was used in three, and 150 mg (132 patients) in one; placebo was given to 689 patients. We used doses of 300 mg, 450 mg, and 600 mg in our pooled analysis.

### Weekly pain response rates

**Figure 1:**
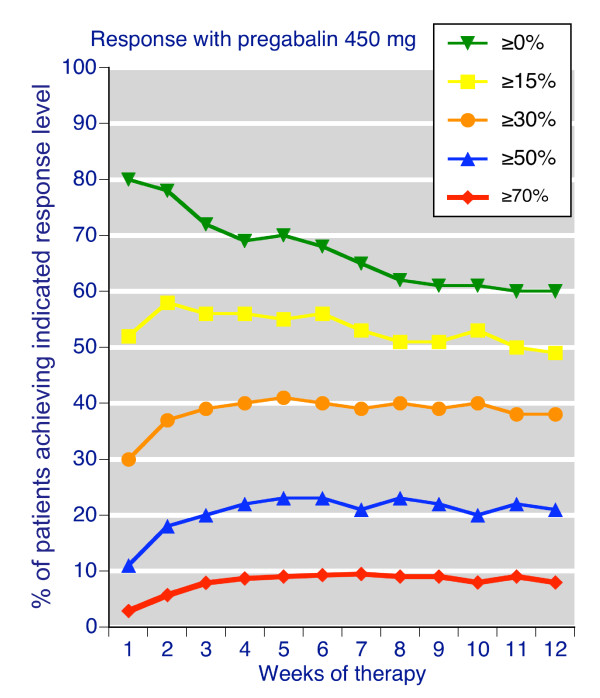
**Weekly pain response levels compared to baseline**. For patients treated with pregabalin 450 mg daily.

Data for weekly pain response with pregabalin 450 mg daily are shown in Figure 1. Additional file 1 compares the weekly pain response with pregabalin 300-600 mg daily and placebo. Numerical data for six and 12 weeks are presented in Table 1. Over time the number of patients reporting 'any improvement' fell and the number reporting the higher response levels of at least 50% or at least 70% improvement increased, demonstrating that change in recorded pain intensity was a sensitive indicator for a responder analysis. This was apparent for placebo and all pregabalin doses, especially over the first six weeks. At 6 weeks the proportion with at least 50% pain relief, a substantial improvement, reached a steady state. After 12 weeks 38% of those treated with 450 mg pregabalin had a moderate response or better, 21% a substantial response, and 8.5% an extensive response.

**Table 1:** Pain and sleep responses at different response levels and doses of pregabalin

		Percent with treatment/placebo				NNT (95% CI)		
		
Outcome	Level	Placebo	300 mg	450 mg	600 mg	300 mg	450 mg	600 mg
Pain: change from baseline	≥ 0%	60	65	68	60	not calculated	13 (8.0 to 41)	not calculated
6 weeks	≥ 15%	44	52	56	52	13 (7.8 to 43)	8.4 (5.8 to 15)	14 (7.6 to 67)
	≥ 30%	28	36	39	39	10 (6.9 to 21)	8.0 (5.7 to 13)	8.5 (5.8 to 16)
	≥ 50%	14	21	23	26	14 (8.8 to 30)	12 (7.9 to 22)	8.8 (6.2 to 15)
	≥ 70%	4.1	7.7	9.0	12	30 (17 to 110)	19 (13 to 38)	14 (9.8 to 26)

Pain: change from baseline	≥ 0%	54	55	60	53	not calculated	not calculated	not calculated
12 weeks	≥ 15%	40	45	49	43	not calculated	12 (6.9 to 35)	not calculated
	≥ 30%	29	33	38	34	not calculated	11 (6.9 to 29)	not calculated
	≥ 50%	15	19	21	23	22 (11 to 870)	16 (9.3 to 59)	13 (8.1 to 31)
	≥ 70%	5.9	6.8	8.5	12	not calculated	not calculated	18 (11 to 43)

Sleep: change from baseline	≥ 0%	59	64	68	61	not calculated	11 (7.0 to 23)	not calculated
6 weeks	≥ 15%	45	53	55	51	11 (7.2 to 29)	9.3 (6.3 to 18)	15 (8.0 to 130)
	≥ 30%	29	39	43	41	9.4 (6.4 to 17)	6.8 (5.1 to 10)	8.2 (5.7 to 15)
	≥ 50%	13	25	26	26	9.0 (6.6 to 14)	7.8 (5.9 to 12)	7.7 (5.7 to 12)
	≥ 70%	3.8	11	13	13	14 (10 to 23)	11 (8.6 to 17)	12 (8.8 to 21)

Sleep: change from baseline	≥ 0%	51	54	58	52	not calculated	14 (7.6 to 68)	not calculated
12 weeks	≥ 15%	37	43	49	44	not calculated	8.9 (5.9 to 19)	16 (8.3 to 170)
	≥ 30%	25	32	40	35	14 (8.1 to 58)	7.0 (5.1 to 11)	10 (6.6 to 22)
	≥ 50%	14	21	26	26	13 (8.2 to 30)	8.4 (6.0 to 14)	8.4 (6.1 to 14)
	≥ 70%	5.0	9.3	10	12	24 (14 to 82)	20 (13 to 55)	14 (9.4 to 24)

NNTs were not calculated when statistical significance was not achieved.

The corresponding NNTs (Table 1, Additional file 2) generally increased over time for all response levels. At 12 weeks, 11 people need to be treated with pregabalin 450 mg daily rather than with placebo for one of them to achieve a moderate benefit of at least 30% pain relief.

### Weekly sleep response rates

**Figure 2:**
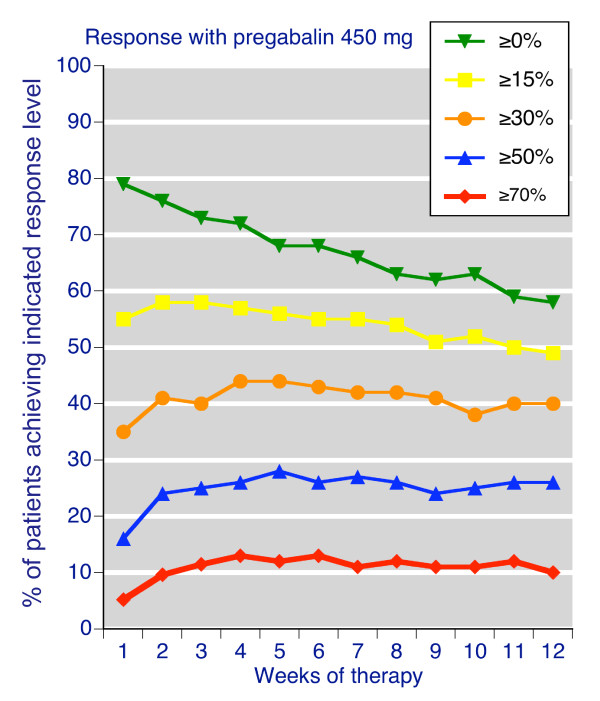
**Weekly sleep response levels compared to baseline**. For patients treated with pregabalin 450 mg daily.

Figure 2 and Additional files 3 and 4 illustrate the percentages of patients achieving the indicated response levels for sleep improvement over time and the corresponding NNTs. The results for sleep response were similar to pain relief, demonstrating that change in sleep was a sensitive indicator for a responder analysis. After 12 weeks with 450 mg pregabalin daily 40% had ≥ 30% improvement, 26% had ≥ 50% improvement, and 10% had ≥ 70% improvement (Table 1).

The corresponding NNTs (Table 1, Additional file 4) generally increased over time for all response levels. At 12 weeks, 7 people need to be treated with pregabalin 450 mg daily rather than with placebo for one of them to achieve a moderate benefit of at least 30% reduction in sleep interference.

### Patient Global Impression of Change

**Figure 3:**
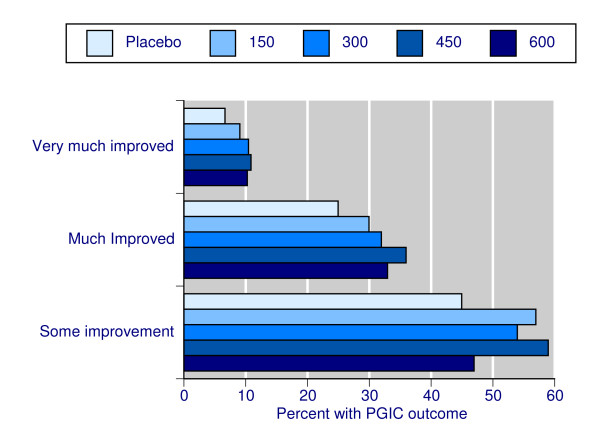
**Patient Global Impression of Change**. The proportion of patients achieving a rating of at least some improvement, at least much improved, or very much improved.

Figure 3 shows the proportion of patients achieving a PGIC rating of very much improved, at least much improved, or at least some improvement at end of study. For the higher hurdles of improvement (much and very much improved), pregabalin was more effective than placebo and a dose response was apparent, although 600 mg daily produced slightly lower levels of improvement than 450 mg. Using 'any improvement' as a measure of efficacy, no consistent and convincing benefit of pregabalin over placebo was apparent. This demonstrates that Patient Global Impression of Change was a sensitive indicator for a responder analysis. NNTs and actual values are shown in Table 2; best sensitivity was shown with 450 mg and the cumulative outcome of much and very much improved.

**Table 2:** PGIC responses at end of study

	Percent with treatment/placebo				NNT (95% CI)		
	
Improvement	Placebo	300 mg	450 mg	600 mg	300 mg	450 mg	600 mg
At least minimal	45	54	59	47	11 (7.1 to 28)	7.3 (5.3 to 12)	not calculated
At least much	25	32	36	33	14 (8.5 to 44)	8.9 (6.2 to 16)	13 (7.7 to 41)
Very much	6.7	11	11	10	26 (15 to >100)	24 (14 to 80)	not calculated

### Other outcomes

Additional file 5 shows responder analyses for a number of other outcomes, including the MAF global fatigue index, FIQ, and HADS depression and anxiety scores, as well as individual domains of the general health status measure SF-36.

Most of these demonstrated sensitivity, in that the proportion of responders fell with increasing levels of response, though this was less marked with some of the individual domains of SF-36, particularly physical and emotional role limitations, social functioning, bodily pain, and vitality. For these the differential between lowest and highest levels of response was not large. Sensitivity to detect an effect of pregabalin treatment defined by statistical significance over placebo to enable NNT to be calculated was apparent for MOS Sleep Disturbance, MOS Sleep Problems Index, and SF-36 general health perception, bodily pain, and vitality.

## Discussion

Analyses presented here involved 2,757 patients with ACR-defined fibromyalgia investigated in high quality randomised double blind trials for eight to 14 weeks. This represents the largest body of evidence available in fibromyalgia, more than double the number of patients investigated in three trials of duloxetine, [16] and four times that with amitriptyline [32]. Moreover, analyses involved a large number of different measures at five different levels of efficacy.

The principal findings were that simple outcomes like pain, sleep, and PGIC were amenable to responder analysis. They demonstrated stepped reductions in value with increasing level of response, showed a significant difference between pregabalin and placebo, and a trend towards lower (better) NNTs at higher doses of pregabalin. With our approach (responder analysis based on percentage change from baseline) this was not generally the case with less simple outcomes, including fatigue, Fibromyalgia Impact Questionnaire scores, anxiety, depression, and most domains of SF-36, apart perhaps vitality. Therefore, responder analysis as performed here is probably not suitable for most of the outcome measures identified in fibromyalgia clinical trials [3].

A minority of patients experience substantial or moderate benefit, though always significantly more than with placebo, whichever IMMPACT definition of benefit is used. Similar levels of response have been seen for duloxetine, amitriptyline, and tramadol/paracetamol in fibromyalgia, [16,32,33] and in osteoarthritis [19].

Weekly analyses for changes in pain intensity and sleep interference demonstrated that maximum benefits for moderate (≥ 30%), substantial (≥ 50%), or extensive (≥ 70%) response occurred at four to six weeks, and thereafter remained reasonably constant. By contrast, response rates for any benefit (≥ 0%) and minimal benefit (≥ 15%) dropped over 12 weeks. Those with a useful response for pain and sleep tend to continue with the treatment; those not achieving moderate or substantial improvement after 4-6 weeks are unlikely to do so later and may be better served by alternative therapies. Pregabalin seemed equally effective at treating pain and sleep disturbance in fibromyalgia, though it is not clear if these improvements occurred in the same patients.

NNTs for reduction in pain intensity and sleep interference calculated at different levels of response at weekly intervals increased with time for all three doses of pregabalin. An increase in NNTs over time has been seen before in arthritis [19]. It may represent either increasing discontinuation rates over time, perhaps because of adverse events with active therapy, or patients who had previously achieved a response at a given level now experiencing a decrease in their magnitude of improvement to below the level in question, or some combination of these. Discontinuations can be different between therapies, with more adverse event discontinuations with active therapy, and more lack of efficacy discontinuations with placebo, and these may have different timescales [34].

Changing NNTs over time are an important finding with implications for efficacy comparisons between drugs. Drugs tested in shorter duration trials (six weeks or less) are likely to appear more effective than the same drug in longer duration trials (eight weeks or more). Four of 10 randomised trials of amitriptyline in fibromyalgia were of six weeks or less, [32] though those of duloxetine were of 12 weeks duration, [16] as was that of a tramadol/paracetamol combination [33].

For the PGIC rating at the end of the trial, higher levels of improvement showed pregabalin to be progressively less effective, at least when NNTs were considered. This illustrates the problem with using 'any improvement' as an outcome, as has been the case in many neuropathic pain studies in the past. Use of 'any improvement' as an outcome overestimated efficacy compared with more substantial levels of improvement.

Table 2 shows that PGIC response rates for 'improvement' decreased at 600 mg pregabalin compared with the 450 mg dose. Perhaps 450 mg is the optimal treatment dose for fibromyalgia (as PGIC takes therapeutic efficacy and adverse events into account). However, it has to be kept in mind that the dose of 450 mg pregabalin was used in all four trials (687 patients) while 600 mg pregabalin was used in only three of them (564 patients). Some inter-trial variability may therefore potentially also play a role.

The strengths of our analysis are that we analysed a large number of individual patient data in a clinically important chronic pain condition, using validated instruments for measuring clinically important trial outcomes, based on large, modern, rigorous, and methodologically sound trials. Our approach is limited in that we have analysed individual patient data for the drug treatment of fibromyalgia for only one agent (pregabalin). More individual patient analyses with other treatments for fibromyalgia are needed to confirm that our findings are generalisable. Finally, any work on fibromyalgia as it is presently defined is limited because 'fibromyalgia' is probably a heterogeneous group of clinical entities with multifaceted patterns of pain, driven by complex pathways of neural mechanisms in which different pathways and mechanisms are not clearly correlated with different pain patterns, likely to be different between individuals, and further complicated by co-morbid conditions and increased age. Chronic pain is associated with functional, structural, and chemical changes in the brain, including loss of gray matter [35]. Individual variability in physiological response to analgesic drugs may be genetic, as for NSAIDs, [36] opioids, [37] and more generally, [38] and indeed varies in extent between different conditions, as with pregabalin in peripheral neuropathic pain, central neuropathic pain, and fibromyalgia [39]. Ongoing genetic, neurobiological, and biomarker work in fibromyalgia [1,2] may one day help to classify patients more appropriately and allow targeted treatment.

## Conclusions

Quite large differences in response levels between individuals with fibromyalgia are to be expected, and were found in this analysis, where responder rates with pregabalin were higher than with placebo. Responder analysis in fibromyalgia looks promising. However, responder analysis in the form that we have undertaken in this paper (using percentage change from baseline) is appropriate only for certain outcomes (such as pain and sleep) and not for others; it is informative where it works but not universally applicable. The full potential and limitations of responder analysis will be realised only when more data can be analysed and compared.

## List of abbreviations

ACR: American College of Rheumatology; CI: confidence interval; NNT: number needed to treat; NSAID: non-steroidal anti-inflammatory drug; RCT: randomised controlled trial; VAS: visual analogue scale; HADS: Hospital Anxiety and Depression Scale; FIQ: Fibromyalgia Impact Questionnaire; SF-36: Short Form 36; MAF: Multidimensional Assessment of Fatigue; PGIC: Patient Global Impression of Change; MOS: Medical Outcomes Study

## Competing interests

SS has received research support and grants from Bandolier, Reckitt Benckiser, and Georg-August-University Göttingen. RAM and HJM have received research grants, consulting, or lecture fees from pharmaceutical companies, including Pfizer, MSD, GSK, AstraZeneca, Grünenthal, Menarini, Futura, and others. RAM and SD have also received research support from charities and government sources at various times. RAM is the guarantor. No author has any direct stock holding in any pharmaceutical company.

## Authors' contributions

RAM and SS were involved with the original concept, planning the study, writing it, analysis, and preparing a manuscript; JP performed the calculation; SD and HJM were involved with planning, and writing. All authors read and approved the final manuscript.

## Pre-publication history

The pre-publication history for this paper can be accessed here:

http://www.biomedcentral.com/1471-2474/11/150/prepub

## Supplementary Material

Additional file 1 **Weekly pain response levels compared to baseline**. This PDF file illustrates pain relief in patients treated with pregabalin at doses of 300-600 mg or placebo. Click here for file

Additional file 2 **NNTs for weekly pain response levels**. This Excel file compares pregabalin at doses of 300-600 mg to placebo. Click here for file

Additional file 3 **Weekly sleep response levels compared to baseline**. This PDF file illustrates sleep response in patients treated with pregabalin at doses of 300-600 mg or placebo. Click here for file

Additional file 4 **NNTs for weekly sleep response levels**. This Excel file compares pregabalin at doses of 300-600 mg to placebo. Click here for file

Additional file 5 **End of trial responder analyses for other measures**. This Excel file details the effects of using different levels of response on a variety of outcomes, including fatigue, sleep, depression, anxiety, and the various domains of SF-36. Click here for file

## References

[B1] Harris RE Clauw DJ How do we know that the pain in fibromyalgia is "real"? Curr Pain Headache Rep 2006 10 403 407 10.1007/s11916-006-0069-0 17087863

[B2] Dadabhoy D Crofford LJ Spaeth M Russell IJ Clauw DJ Biology and therapy of fibromyalgia Evidence-based biomarkers for fibromyalgia syndrome Arthritis Res Ther 2008 10 211 10.1186/ar2443 18768089 PMC2575617

[B3] Carville SF Choy EH Systematic review of discriminating power of outcome measures used in clinical trials of fibromyalgia J Rheumatol 2008 35 2094 2105 10.3899/jrheum.080077 18792996

[B4] Wolfe F Smythe HA Yunus MB Bennett RM Bombardier C Goldenberg DL Tugwell P Campbell SM Abeles M Clark P Fam AG Farber SJ Fiechtner JJ Franklin CM Gatter RA Hamaty D Lessard J Lichtbroun AS Masi AT Mccain GA Reynolds WJ Romano TJ Russell IJ Sheon RP The American College of Rheumatology 1990 Criteria for the Classification of Fibromyalgia. Report of the Multicenter Criteria Committee Arthritis Rheum 1990 33 160 172 10.1002/art.1780330203 2306288

[B5] Russell IJ Raphael KG Fibromyalgia syndrome: presentation, diagnosis, differential diagnosis, and vulnerability CNS Spectr 2008 13 6 11 18323767 10.1017/s1092852900026778

[B6] McNalley JD Matheson DA Bakowshy VS The epidemiology of self-reported fibromyalgia in Canada Chronic Dis Can 2006 27 9 16 16672135

[B7] Mas AJ Carmona L Valverde M Ribas B EPISER Study Group Prevalence and impact of fibromyalgia on function and quality of life in individuals from the general population: results from a nationwide study in Spain Clin Exp Rheumatol 2008 26 519 526 18799079

[B8] Bannwarth B Blotman F Roué-Le Lay K Caubère JP André E Taïeb C Fibromyalgia syndrome in the general population of France: A prevalence study Joint Bone Spine 2009 76 184 187 10.1016/j.jbspin.2008.06.002 18819831

[B9] Hawley DJ Wolfe F Pain, disability, and pain/disability relationships in seven rheumatic disorders: a study of 1,522 patients J Rheumatol 1991 18 1552 1557 1837315

[B10] Martinez JE Ferraz MB Sato EI Atra E Fibromyalgia versus rheumatoid arthritis: a longitudinal comparison of the quality of life J Rheumatol 1995 22 270 274 7738950

[B11] Christakis NA Does this work for you? BMJ 2008 337 a2281 10.1136/bmj.a2281

[B12] Moore A Straube S Derry S McQuay H Individuals, averages, and evidence based medicine BMJ 2008 337 a2585 10.1136/bmj.a2585 19019866

[B13] Moore RA Edwards JE McQuay HJ Acute pain: individual patient meta-analysis shows the impact of different ways of analysing and presenting results Pain 2005 116 322 331 10.1016/j.pain.2005.05.001 15979792

[B14] Finnerup NB Otto M McQuay HJ Jensen TS Sindrup SH Algorithm for neuropathic pain treatment: an evidence based proposal Pain 2005 118 289 305 10.1016/j.pain.2005.08.013 16213659

[B15] Straube S Derry S McQuay HJ Moore RA Enriched enrolment: definition and effects of enrichment and dose in trials of pregabalin and gabapentin in neuropathic pain A systematic review Br J Clin Pharmacol 2008 66 266 275 10.1111/j.1365-2125.2008.03200.x 18489611 PMC2492925

[B16] Sultan A Gaskell H Derry S Moore RA Duloxetine for painful diabetic neuropathy and fibromyalgia pain: systematic review of randomised trials BMC Neurol 2008 8 29 10.1186/1471-2377-8-29 18673529 PMC2529342

[B17] Dahlof CG Pascual J Dodick DW Dowson AJ Efficacy, speed of action and tolerability of almotriptan in the acute treatment of migraine: pooled individual patient data from four randomized, double-blind, placebo-controlled clinical trials Cephalalgia 2006 26 400 408 10.1111/j.1468-2982.2005.01080.x 16556240

[B18] Moore RA Moore OA Derry S McQuay HJ Numbers needed to treat calculated from responder rates give a better indication of efficacy in osteoarthritis trials than mean pain scores Arthritis Res Ther 2008 10 R39 10.1186/ar2394 18384679 PMC2453757

[B19] Moore RA Moore OA Derry S Peloso PM Gammaitoni AR Wang H Responder analysis for pain relief and numbers needed to treat in a meta-analysis of etoricoxib osteoarthritis trials: bridging a gap between clinical trials and clinical practice Ann Rheum Dis 2010 69 374 379 10.1136/ard.2009.107805 19364730 PMC2800200

[B20] Dworkin RH Turk DC Wyrwich KW Beaton D Cleeland CS Farrar JT Haythornthwaite JA Jensen MP Kerns RD Ader DN Brandenburg N Burke LB Cella D Chandler J Cowan P Dimitrova R Dionne R Hertz S Jadad AR Katz NP Kehlet H Kramer LD Manning DC McCormick C McDermott MP McQuay HJ Patel S Porter L Quessy S Rappaport BA Rauschkolb C Revicki DA Rothman M Schmader KE Stacey BR Stauffer JW von Stein T White RE Witter J Zavisic S Interpreting the clinical importance of treatment outcomes in chronic pain clinical trials: IMMPACT recommendations J Pain 2008 9 105 121 10.1016/j.jpain.2007.09.005 18055266

[B21] Crofford LJ Rowbotham MC Mease PJ Russell IJ Dworkin RH Corbin AE Young JP Jr LaMoreaux LK Martin SA Sharma U Pregabalin 1008-105 Study Group Pregabalin for the treatment of fibromyalgia syndrome: results of a randomized, double-blind, placebo-controlled trial Arthritis Rheum 2005 52 1264 1273 10.1002/art.20983 15818684

[B22] Mease PJ Russell IJ Arnold LM Florian H Young JP Jr Martin SA Sharma U A randomized, double-blind, placebo-controlled, phase III trial of pregabalin in the treatment of patients with fibromyalgia J Rheumatol 2008 35 502 514 18278830

[B23] Arnold LM Russell IJ Diri EW Duan WR Young JP Jr Sharma U Martin SA Barrett JA Haig G A 14-week, randomized, double-blinded, placebo-controlled monotherapy trial of pregabalin in patients with fibromyalgia J Pain 2008 9 792 805 10.1016/j.jpain.2008.03.013 18524684

[B24] Pauer L Danneskiold-Samsoe B Jespersen A Pregabalin for the management of Fibromyalgia (FM): A 14-week, randomised, double-blind, placebo controlled, monotherapy trial (Study A0081100) Ann Rheum Dis 2008 67 Suppl 25617604285

[B25] Crofford LJ Mease PJ Simpson SL Young JP Jr Martin SA Haig GM Sharma U Fibromyalgia relapse evaluation and efficacy for durability of meaningful relief (FREEDOM): a 6-month, double-blind, placebo-controlled trial with pregabalin Pain 2008 136 419 431 10.1016/j.pain.2008.02.027 18400400

[B26] McQuay HJ Derry S Moore RA Poulain P Legout V Enriched enrolment with randomised withdrawal (EERW): Time for a new look at clinical trial design in chronic pain Pain 2008 135 217 220 10.1016/j.pain.2008.01.014 18258369

[B27] Jadad AR Moore RA Carroll D Jenkinson C Reynolds DJ Gavaghan DJ McQuay HJ Assessing the quality of reports of randomized clinical trials: is blinding necessary? Control Clin Trials 1996 17 1 12 10.1016/0197-2456(95)00134-4 8721797

[B28] Smith LA Oldman AD McQuay HJ Moore RA Teasing apart quality and validity in systematic reviews: an example from acupuncture trials in chronic neck and back pain Pain 2000 86 119 132 10.1016/S0304-3959(00)00234-7 10779669

[B29] Cook RJ Sackett DL The number needed to treat: a clinically useful measure of treatment effect BMJ 1995 310 452 454 7873954 10.1136/bmj.310.6977.452 PMC2548824

[B30] Moore RA Gavaghan D Tramèr MR Collins SL McQuay HJ Size is everything--large amounts of information are needed to overcome random effects in estimating direction and magnitude of treatment effects Pain 1998 8 209 216 10.1016/S0304-3959(98)00140-7 9870574

[B31] Straube S Derry S Moore RA McQuay HJ Pregabalin in fibromyalgia: meta-analysis of efficacy and safety from company clinical trial reports Rheumatology (Oxford) 2010 49 706 715 10.1093/rheumatology/kep432 20056767

[B32] Nishishinya B Urrútia G Walitt B Rodriguez A Bonfill X Alegre C Darko G Amitriptyline in the treatment of fibromyalgia: a systematic review of its efficacy Rheumatology (Oxford) 2008 47 1741 1746 10.1093/rheumatology/ken317 18697829

[B33] Bennett RM Kamin M Karim R Rosenthal N Tramadol and acetaminophen combination tablets in the treatment of fibromyalgia pain: a double-blind, randomized, placebo-controlled study Am J Med 2003 114 537 545 10.1016/S0002-9343(03)00116-5 12753877

[B34] Moore RA Derry S McQuay HJ Discontinuation rates in clinical trials in musculoskeletal pain: meta-analysis from etoricoxib clinical trial reports Arthritis Res Ther 2008 10 R53 10.1186/ar2422 18466615 PMC2483442

[B35] Tracey I Bushnell MC How neuroimaging studies have challenged us to rethink: is chronic pain a disease? J Pain 2009 10 1113 1120 10.1016/j.jpain.2009.09.001 19878862

[B36] Fries S Grosser T Price TS Lawson JA Kapoor S DeMarco S Pletcher MT Wiltshire T FitzGerald GA Marked interindividual variability in the response to selective inhibitors of cyclooxygenase-2 Gastroenterology 2006 130 55 64 10.1053/j.gastro.2005.10.002 16401468

[B37] Klepstad P Dale O Skorpen F Borchgrevink PC Kaasa S Genetic variability and clinical efficacy of morphine Acta Anaesthesiol Scand 2005 49 902 908 10.1111/j.1399-6576.2005.00772.x 16045647

[B38] Lötsch J Geisslinger G Current evidence for a genetic modulation of the response to analgesics Pain 2006 121 1 5 10.1016/j.pain.2006.01.010 16472919

[B39] Moore RA Straube S Wiffen PJ Derry S McQuay HJ Pregabalin for acute and chronic pain in adults Cochrane Database Syst Rev 2009 3 CD007076 19588419 10.1002/14651858.CD007076.pub2 PMC4167351

