# Supplementary figures and images for: Pregabalin in fibromyalgia - responder analysis from individual patient data
Source: BMC Musculoskelet Disord. 2010 Jul 5;11:150. doi: 10.1186/1471-2474-11-150 (PMC2906437; doi:10.1186/1471-2474-11-150)

Additional file 1: Weekly pain response levels compared to baseline

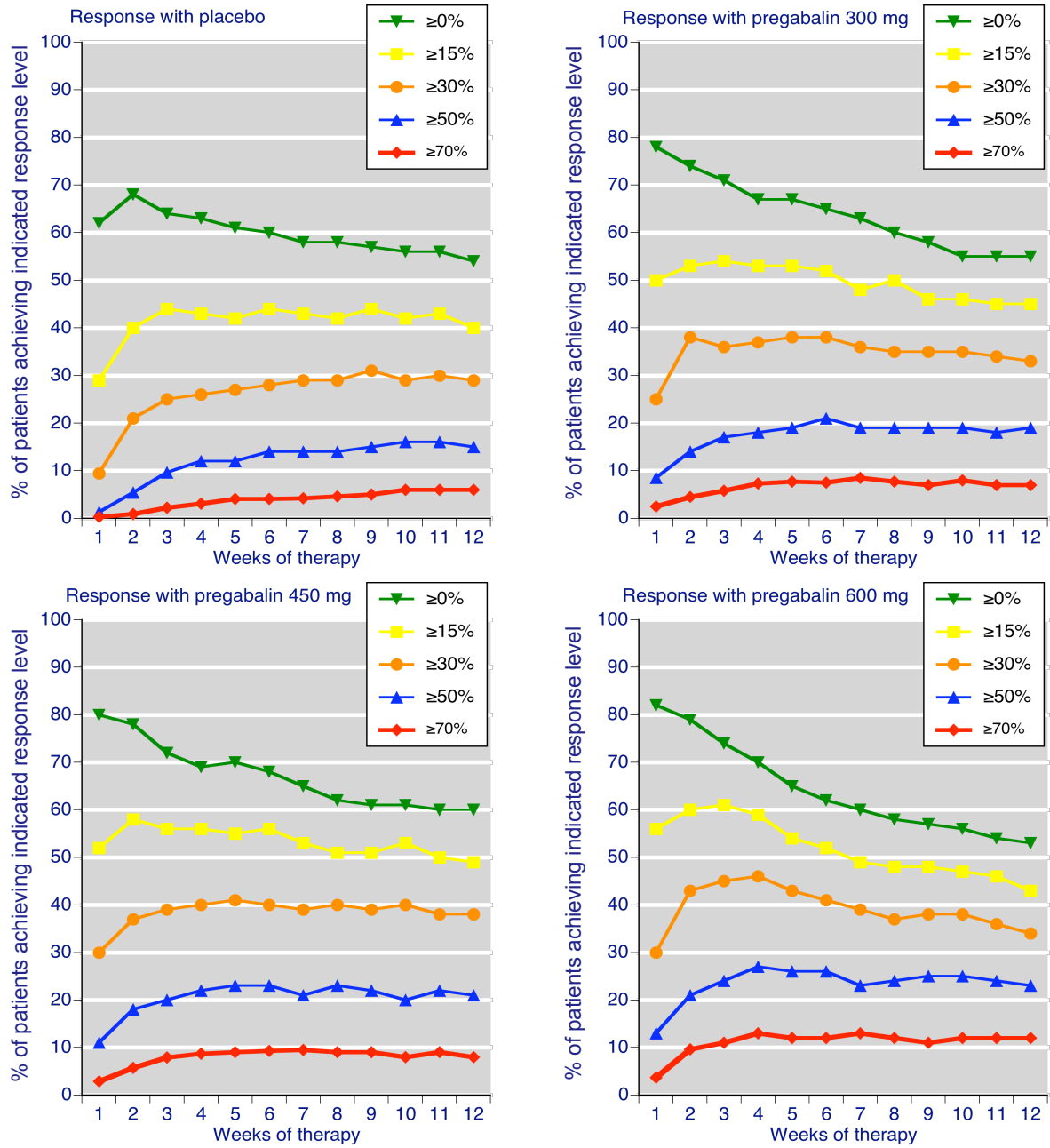

Supplement: Additional file 1 — Weekly pain response levels compared to baseline. This PDF file illustrates pain relief in patients treated with pregabalin at doses of 300-600 mg or placebo. [file 1471-2474-11-150-S1.PDF]

### Additional file 3: Weekly sleep response levels compared to baseline

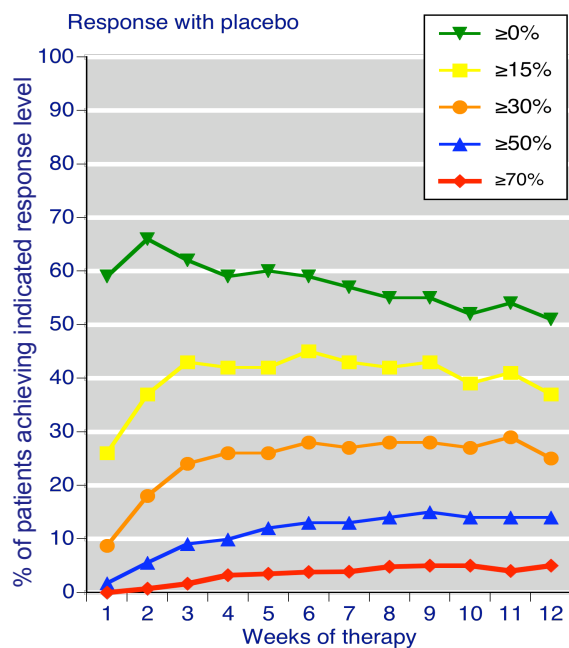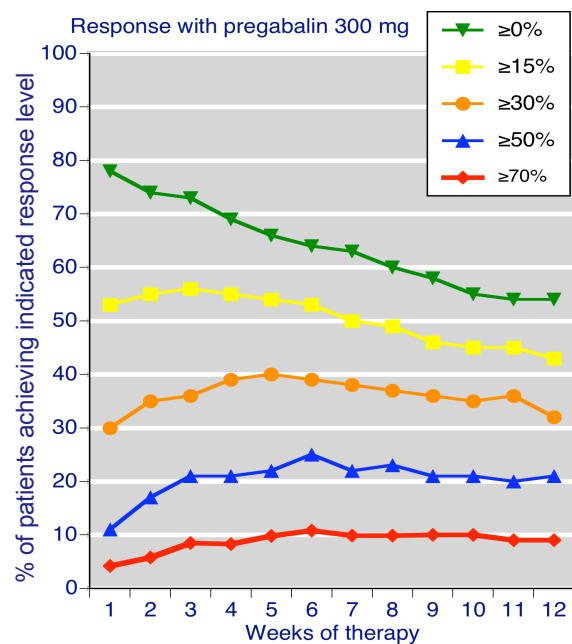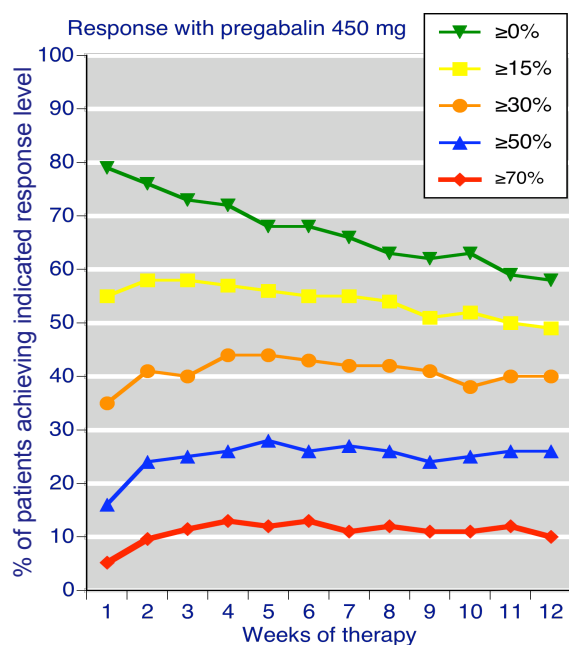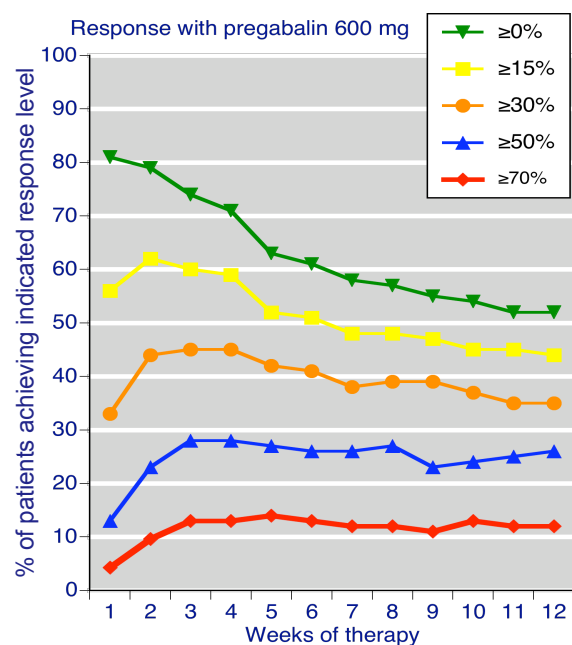

Supplement: Additional file 3 — Weekly sleep response levels compared to baseline. This PDF file illustrates sleep response in patients treated with pregabalin at doses of 300-600 mg or placebo. [file 1471-2474-11-150-S3.PDF]
